# Classic ataxia-telangiectasia: the phenotype of long-term survivors

**DOI:** 10.1007/s00415-019-09641-1

**Published:** 2019-11-27

**Authors:** Nienke J. H. van Os, Marcel van Deuren, Corry M. R. Weemaes, Judith van Gaalen, Helma Hijdra, Alexander M. R. Taylor, Bart P. C. van de Warrenburg, Michèl A. A. P. Willemsen

**Affiliations:** 1 Department of Pediatric Neurology, Radboudumc Amalia Children’s Hospital, Donders Institute for Brain, Cognition and Behaviour, Radboud University Medical Center, Nijmegen, The Netherlands; 2 Department of Neurology, Donders Institute for Brain, Cognition and Behaviour, Radboud University Medical Center, Nijmegen, The Netherlands; 3 Department of Internal Medicine, Radboud Institute for Molecular Life Sciences, Radboud University Medical Center, Nijmegen, The Netherlands; 4 Department of Pediatrics, Pediatric Infectious Disease and Immunology, Radboudumc Amalia Children’s Hospital, Radboud University Medical Center, Nijmegen, The Netherlands; 5 Department of Rehabilitation Medicine, Radboud University Medical Center, Nijmegen, The Netherlands; 6 Institute of Cancer and Genomic Sciences, University of Birmingham, Birmingham, United Kingdom

**Keywords:** Ataxia–telangiectasia, Louis–Bar syndrome, *ATM* gene, Long-survivors, Prognosis

## Abstract

**Objective:**

Patients with classic ataxia–telangiectasia (A–T) generally die in the second or third decade of life. Clinical descriptions of A–T tend to focus on the symptoms at presentation. However, during the course of the disease, other symptoms and complications emerge. As long-term survivors with classic A–T develop a complex multisystem disorder with a largely unknown extent and severity, we aimed to comprehensively assess their full clinical picture.

**Methods:**

Data from Dutch patients with classic A–T above the age of 30 years were retrospectively collected. In addition, we searched the literature for descriptions of classic A–T patients who survived beyond the age of 30 years.

**Results:**

In the Dutch cohort, seven classic A–T patients survived beyond 30 years of age. Fourteen additional patients were retrieved by the literature search. Common problems in older patients with classic A–T were linked to ageing. Most patients had pulmonary, endocrine, cardiovascular, and gastro-intestinal problems. All patients had a tetraparesis with contractures. This led to immobilization and frequent hospital admissions. Most patients expressed the wish to no longer undergo intensive medical treatments, and waived follow-up programs.

**Conclusions:**

Paucity of descriptions in the literature, and withdrawal from medical care complicate the acquisition of follow-up data on the natural history of long-term survivors. Irrespective of these limitations, we have obtained impression of the many problems that these patients face when surviving beyond 30 years of age. Awareness of these problems is needed to guide follow-up, counselling, and (palliative) care; decisions about life-prolonging treatments should be well considered.

**Electronic supplementary material:**

The online version of this article (10.1007/s00415-019-09641-1) contains supplementary material, which is available to authorized users.

## Introduction

Ataxia telangiectasia (A–T) is a neurodegenerative disorder caused by mutations in the *ATM* gene, coding for an enzyme that plays a role in cell cycle control and DNA repair [1]. A–T is characterized by cerebellar ataxia, extrapyramidal movement disorders, oculocutaneous telangiectasia, immunodeficiency and pulmonary dysfunction [2]. Due to the underlying DNA repair defect, patients are radiosensitive and at increased risk of cancer [3]. Most patients become wheelchair bound around the age of 10 years [2] and generally die in the second or third decade of life due to malignancies or respiratory insufficiency [4–6].

Some A–T patients survive beyond the age of 30 years. Most of them have ‘variant A-T’ and their prolonged survival results from the presence of some residual ATM kinase activity [4, 7]. These patients have a milder neurological disease course with absence of immunodeficiency and pulmonary problems [7, 8]. A small proportion of A–T patients who survive beyond the age of 30 years, however, lack ATM kinase activity and have classic A–T. These patients are labelled ‘long-term survivors’, representing exceptional cases in a group with a severely reduced life-expectancy. Detailed descriptions of long-term survivors with classic A–T are rare and were never studied systematically. Importantly, improvements in symptomatic treatment of patients with A–T may lead to an increase in survival. Therefore, the problems that older patients with A-T are confronted with, need to be recognized. Here we aim to comprehensively define the phenotype of long-term survivors with classic A–T by combining data from the Dutch cohort with a review of the literature.

## Methods

### Dutch cohort

Clinical, genetic and laboratory data of classic A–T patients who survived beyond the age of 30 years were retrospectively collected from their medical records and from our database of genetically confirmed A–T cases. All Dutch patients had the classic A–T phenotype as described in the “Introduction” and all but two had demonstrated absence of residual ATM kinase activity. The remaining two siblings were compound heterozygous for a frameshift mutation and a nonsense mutation, respectively, that would both prevent expression of any ATM protein. As expected from the presence of these mutations they had a classic phenotype as previously described [4].

### Literature search

We searched the PubMed database for reports on patients with classic A–T beyond the age of 30 years. We combined search terms for ‘ataxia telangiectasia’ (Louis–Bar syndrome), with search terms for ‘old’ (adult*, long, surviv*, elder*, matur*, senior) or ‘classic’ (classic*). Patients with a mild, atypical or variant phenotype (other than only a long-term survival) and patients with proven residual ATM kinase activity were excluded. Additional reports were identified through hand-searching of reference lists of relevant papers.

### Extraction of data

The following data were retrieved from the patients’ medical records or our database: sex, age, age of death, cause of death, *ATM* mutations, and results from studies in lymphoblastoid cell lines (i.e., expression of ATM protein and absence of residual ATM kinase activity). For patients who were annually seen at our centre, medical records were searched for medical history, prescribed medication, and problems mentioned during interviews. Abnormal findings at physical examination and from laboratory and other diagnostic tests were collected. Furthermore, medical histories and our database were specifically queried for diseases, abnormalities or problems that were found in other patients or in the literature, in an iterative manner. For deceased patients who were not annually followed at our centre, data were retrieved from the patients’ primary medical centre. If mentioned, the same data were extracted for the patients described in the literature. If data were not reported, these were noted as ‘missing’ instead of ‘not present’. The only exception we made was for malignancy: if its presence was not reported in a detailed case description, we assumed this was not present.

## Results

The Dutch cohort consisted of seven classic A–T patients (four male and three female) who survived beyond the age of 30 years. Their ages ranged from 32 to 54 years. Three patients were alive at the end of follow-up, while four patients had deceased. All seven patients were previously described [4, 7] (see Table 1). A case description with photo- and video material of both the youngest (patient 1, age 32 years) and oldest (patient 7, age 54 years) patient from this series are shown in Online Resources 1 (case descriptions), 2 (photos of patient 1), and 3 (video of patient 7), respectively*.*

**Table 1:** Seven classic A–T patients from the Dutch cohort who survived beyond the age of 30 years

	Patient 1	Patient 2	Patient 3	Patient 4	Patient 5	Patient 6	Patient 7
Described previously	Van Os [4] (24b)	Van Os [4] (24a)	Van Os [4] (23b), Verhagen [7] (11.I)	Van Os [4] (35a), Verhagen [7] (32.II)	Van Os [4] (35b), Verhagen [7] (32.I)	Van Os [4] (11), Verhagen [7] (4)	Van Os [4] (13)
Sex	F	M	M	F	F	M	M
Age	32	37	38	49^†,^^a^	52^†^	40^†^	54^†^
Cause of death	–	–	–	Sepsis	Cardiac arrest	Malignancy	Malignancy
Mutation 1	c.4109 + 5G > A; p.(Asp1371Glyfs*)	c.4109 + 5G > A; p.(Asp1371Glyfs*)	c.3576G > A; p.(Ser1135_Lys1192del58)	c.2839-579_2839-576del4; p.(Met946fs*)	c.2839-579_2839-576del4; p.(Met946fs*)	c.1563_ 1564delGA; p.(Glu522fs)	c.331 + 5G > A; p.(Ala112SerfsX3)
Mutation 2	c.7875_7876delTGinsGC; p.(Asp2625_Ala2626delinsGluPro)	c.7875_7876delTGinsGC; p.(Asp2625_Ala2626delinsGluPro)	c.3576G > A; p.(Ser1135_Lys1192del58)	c.4396C > T; p.(Arg1466*)	c.4396C > T; p.(Arg1466*)	c.3078G > T; p.(Trp1026Cys)	c.4040delT; p.(Leu1347TyrfsX2)
ATM protein	Present	Present	Present	Not tested	Not tested	Absent	Absent
ATM kinase activity	Absent	Absent	Absent	Not tested	Not tested	Absent	Absent

^a^This patient died one week before her 49th birthday

Fourteen additional A–T patients beyond the age of 30 were retrieved by the literature search [9–19] (see Table 2). In only three of them it was mentioned that the diagnosis of A–T was confirmed genetically. In ten patients, the clinical diagnosis of A–T was supported by elevated serum alpha-protein (AFP) levels, and/or the presence of typical cytogenetic abnormalities (see Online Resource 4). In one patient, the clinical diagnosis, which we considered convincing enough to include the patient in the present study, was not substantiated by laboratory results.

**Table 2:** Fourteen classic A–T patients from the literature who survived beyond the age of 30 years

References	Patient	Sex	Age	Cause of death
Goodman [19]	1	M	42	–
	2	F	38	–
Amromin [9]	Single case	F	32^†^	Respiratory failure
Agamanolis [10]	Single case	M	31†	Respiratory failure with cardiac arrest
Cabot [11]	Case 2	M	30†	Malignancy
Mock [12]	Single case	F	34†	Malignancy
Kovacs [13]	Case 2	F	34†	Multiple causes
	Case 3	F	38†	Respiratory failure
Opeskin [14]	Single case	M	34†	Respiratory failure
Degan [15]	LA^a^	M	34	–
Habek [16]	Single case	V	34†	Sepsis
Lockman [17]	E^a^	F	31†	Respiratory failure
	G^a^	F	33†	Respiratory failure and sepsis
Lin [18]	9	M	34	–

^a^Genetically confirmed diagnosis

The problems that were found in classic A–T patients beyond the age of 30 years are listed in Table 3. We decided to divide these problems into four categories: general, neurological, functional and psychosocial problems. These results will be discussed in the next section.

**Table 3:** Problems in classic A–T patients beyond the age of 30 years

Category	Problem	*N* in 7 Dutch patients (present/reported)^a^	*N* in 14 literature patients (present/reported)^a^
General	Progeric features of skin and hair	4/4	4/4
	Malignancy	3/7	7/12
	Solid	2/3	2/7
	Haematological	1/3	5/7
Pulmonary	Severe lung infections in adulthood	2/6	6/9
	Restrictive lung disease	4/4	0/0
	Tachypnea and/or tachycardia	7/7	3/3
Immunology	IgG_2_ deficiency	3/6	1/6
	IgA deficiency	2/7	4/6
	High IgM (without IgG and IgA deficiency)	4/7	0/4
	Use of immunoglobulin substitution therapy	0/6	0/0
	Use of antibiotic prophylaxis	0/6	1/1
Endocrine/metabolic	Low body weight and BMI	3/5	5/5
	Hyperthyroidism	2/5	0/1
	(Insulin resistant) diabetes mellitus/elevated glucose or HbA1c levels	6/7	3/4
	Hypercholesterolemia	4/4	0/1
Cardiovascular	Hypertension/cardiomyopathy	4/7	1/1
	Iron deficiency anaemia	5/7	0/1
	Telangiectasia in other organs than eyes/skin	1/4	6/6
Gastro-intestinal	Gastro-oesophageal reflux	3/6	1/1
	Elevated liver function tests	5/7	2/4
	Hepatic steatosis	3/3	1/2
	Enlarged liver	2/5	2/2
Neurological	Generalized muscle weakness causing tetraparesis/paralysis with anteflexion of head	7/7	8/8
	Peripheral neuropathy with atrophy, areflexia, autonomic dysfunction	5/5	8/8
	Severe dysarthria/anarthria	7/7	8/9
	Contractures/deformities	6/6	5/5
	Scoliosis	1/4	4/4
	Oculomotor apraxia	6/7	7/7
Functional	Immobility (wheelchair)	7/7	8/8
	ADL dependency	7/7	4/4
	Urine incontinence	3/5	0/0
	Constipation/haemorrhoids	4/7	0/0
	Fractures/osteoporosis	3/3	0/0
	Pressure sores	5/6	0/0
Psychosocial	Pain	2/5	0/0
	Mood problems	3/4	0/0
	Cognitive problems	1/5	4/7
	Fatigue	3/5	0/0
	Recurrent admissions to hospital	4/7	3/3
	Parents are caretakers	5/7	0/0
	Withdrawal from medical care/wish for treatment limitation	6/7	2/2

^a^For some patients data were missing (i.e., not known, not noted or not tested for)

## Discussion

A–T is generally considered to be a childhood disease since the majority of patients present in early life and die in or before early adulthood. However, some patients survive longer than would be expected. Their clinical picture includes a combination of medical, psychosocial and existential problems.

### General problems

The number of classic A–T patients beyond the age of 30 years who developed a malignancy seems to be somewhat higher compared to percentages found in larger A–T cohort studies [20, 21], probably due to the longer follow-up duration. Survival after the development of a malignancy is short in A–T [4], at least partly due to the fact that radiation and chemotherapy cannot be applied at appropriate doses due to the underlying disease mechanism (DNA repair defect, increased radiosensitivity). Older patients with A–T have the additional disadvantage of a severe restrictive impairment of their lung function, which makes mechanical ventilation, and therefore oncologic surgery, challenging [17, 22].

The presence of tachypnea and tachycardia in older patients with A–T seems to reflect their poor, mainly restrictively disturbed lung function. Severe pulmonary infections occurred frequently in older patients with A–T and were a cause of death in the majority of them [9, 10, 13, 17], due to a combination of a troublesome general condition, impairments of pulmonary function, and severe motor disturbances including dysphagia. The immunodeficiency in older patients with A–T does not seem to progress. Interestingly, three Dutch patients who had an IgG_2_ deficiency managed to survive beyond the age of 30 years, whilst IgG_2_ deficiency is correlated with poor survival in A–T [4]. Apparently, other factors played a role in these cases. IgM was asymptomatically elevated in four Dutch patients. None of them had a hyper-IgM phenotype with IgA and IgG deficiency, a phenotype that is associated with a severe reduction in life expectancy [4, 23].

Diabetes mellitus is a known problem in older patients with A–T [24, 25]. Two Dutch sisters had diabetes mellitus with severe insulin resistance, complicated by retinopathy and nephropathy. The presence of diabetes mellitus in older patients with A–T, combined with a relatively high number of older A–T patients with hypercholesterolemia and hypertension, show that metabolic syndrome is a major age-related problem in A–T [25]. One Dutch patient had an ischemic stroke at the age of 37 years. To our knowledge, ischemic stroke—in contrast to intracerebral haemorrhage due to brain telangiectasias or tumours—was never reported before in A–T.

Our data confirm that elevated serum liver enzymes and hepatic steatosis are frequently encountered in patients with A–T [25–28] and worsen with age [25]. Although progression to liver fibrosis or cirrhosis is described in the literature [9, 25, 26], we did not encounter clinically relevant liver function problems in our patients.

Interestingly, five Dutch patients developed an iron deficiency anaemia. Further diagnostic investigations were conducted in only one patient (patient 7, see Online Appendix e-1). Nevertheless, all patients received iron supplementation. This phenomenon was not previously described in A–T, but unexplained anaemia is a common phenomenon in the elderly [29]. During life, telangiectasias expand from sclerae and skin to other organs within the body of A–T patients, the brain being the most frequently documented one [9, 10, 13, 14, 16, 17].

### Neurological problems

The neurological phenotype in older patients with A–T is mainly characterized by a progressive neuropathy and anterior horn cell disease.[9, 19, 30]. Cerebellar ataxia and extrapyramidal movement disorders such as chorea, tremor and myoclonus seem to “disappear”, as these (central) movement disorders are superseded by the loss of motor function due to progressive neuromuscular involvement. Due to a combination of muscle weakness and dystonia, most older patients have difficulties raising their head (so-called ‘head drop’), causing neck pain and problems with feeding and talking. As the (originally mainly cerebellar and dystonic) dysarthria worsens due to muscle weakness and hypophonia, the speech becomes less intelligible. Bone deformities, joint contractures and scoliosis were common in older patients with A–T.

### Functional problems

All patients with classic A–T beyond the age of 30 years were wheelchair-bound and dependent on others in activities of daily living (ADL). As a result of immobilization most patients developed pressure sores and had constipation. Furthermore, patients had osteoporosis and were at risk for bone fractures, due to a combination of immobility, poor weight status and endocrine abnormalities [31, 32]. In two Dutch patients, a bone fracture occurred semi-spontaneously (after a minor trauma). Functional impairments in arm and hand functions are difficult to compensate with aids or devices due to the muscle weakness. All Dutch patients were unable to write, type, or use a phone. All patients needed help with feeding. Eye movement abnormalities (such as abnormal saccades and pursuit, and nystagmus) increase with age in A–T, [33] and therefore the use of a visual aid tool to improve visual function is often not an option.

### Psychosocial problems

Although validated questionnaires to assess quality of life were not used by us, nor was this explicitly scored in any of the patients from the literature, we believe this is poor in older patients with A–T. Next to the neurological complications of the disease, patients suffered from pain due to contractures and from mood disturbances. Probably the awareness of their incapacities and functional limitations increased with age [34]. In none of the patients the cognitive functions were formally tested. However, some patients were stated to have a cognitive impairment. We believe their cognition was partly underestimated due to aforementioned motor and speech impairments. It is known that cognitive dysfunction and mood disturbances can be part of the cerebellar cognitive affective syndrome, that emerges with age in A–T [35]. We did not find evidence for the development of overt dementia in A–T.

Fatigue was often reported in older patients with A–T, comparable to other hereditary ataxias [36]. This can partly be explained by the restricted lung function in A–T, causing decreased tissue oxygenation. Another problem was the fact that these older patients have older parents. Regardless of their own age, parents continued to be the most prominent caregivers for their child with A–T. It is generally known that the primary concern of most parents is the health of, and care for, their handicapped child [37]. Confronted with the problems of their child, parents may deny their own health problems [38].

In the last years of their lives, most patients were recurrently admitted to the hospital, mainly because of severe infections or respiratory problems. During these admissions, decisions about life-prolonging treatments often came up, and in most cases, the patients themselves requested for a treatment limitation.

### Strengths and limitations

One of the major limitations of this study is the lack of complete data, what seems inherent to the population studied. Older patients tended to withdraw from medical care and requested limitations in diagnostic procedures since these were too invasive or painful. Because the presence of many health issues was not systematically tested for, we had to deal with a high proportion of missing data, leading to more narrative reviews of some clinical domains. Reports from the literature mainly focused on autopsy or imaging findings in older patients with A–T, and functional and psychosocial problems were heavily underreported.

### Future directions

All Dutch patients presented in this study had absence of residual ATM kinase activity and therefore, they must have another reason for their prolonged survival. Interestingly, all except for one were compound heterozygous for two different *ATM* mutations and thus have non-consanguineous parents, suggesting that this may rescue them from the presence of other autosomal recessive inherited genetic factors that may negatively influence survival. The only Dutch patient with a homozygous *ATM* mutation, harboured the *ATM* c.3576G > A splice site mutation, which was previously suggested to cause a mild phenotype with longer survival [39]. Future studies should try to identify other genetic and environmental factors that may contribute to long-term survival in A–T.

We believe that in older A–T patients, decisions about life-prolonging treatments and chances of recovery should be well considered and discussed within the medical team and with the patients and their families. Furthermore, the care for patients with a rare disease such as A–T should be coordinated by doctors familiar with the disease. We recommend a multidisciplinary team that consists of both paediatric and adult physicians, to simplify and optimize transition of care and to prevent patients from getting lost to follow-up.

## Conclusion

In its most common form, A–T is a severe, multisystem and progressive disease. Long-term survival of classic A–T is accompanied by an increase in both physical as psychosocial problems. Ongoing and structured follow-up of A–T patients, also in the final stage of their disease, is important for prevention, timely identification, and management of these problems.

## Electronic supplementary material

Below is the link to the electronic supplementary material.

Supplementary file1 (PDF 48 kb)

Supplementary file2 (PDF 147 kb)

Supplementary file3 (AVI 55395 kb)

Supplementary file4 (PDF 26 kb)
