# Supplementary material for: Classic ataxia-telangiectasia: the phenotype of long-term survivors
Source: J Neurol. 2019 Nov 27;267(3):830–7. doi: 10.1007/s00415-019-09641-1 (PMC7035236; doi:10.1007/s00415-019-09641-1)
Supplement: Supplementary file 1 — Supplementary file1 (PDF 48 kb) [file 415_2019_9641_MOESM1_ESM.pdf]

Online Resource 1: Case descriptions of patients 1 and 7 from the Dutch cohort.

Patient 1

This female patient has a classic disease course of A-T. Walking difficulties were noted at the 4th year of life and she became wheelchair bound at the age of 10 years. The diagnosis of A-T was genetically confirmed and she had no residual ATM kinase activity. When she was 24 years old, a sinus tachycardia (up till 130/min) was noted, probably due to respiratory problems caused by a severe restrictive lung function, since she had a vital capacity less than 1.5 litre. She developed a tetraparesis and had difficulties raising her head due to weakness of neck extensor muscles and dystonia, which caused pain in her neck and loss of food from her mouth during eating. Botulinum toxin was considered but not applied because of fear for a decline in swallowing function.

She developed a scoliosis and painful contractures in her feet, knees, elbows, wrists and fingers. Splints were not endured due to pain. Her hands converted to a claw position with a painful red, shiny and tight skin (See *Online Resource 2*). Elective surgery could only be conducted under general anaesthesia, which was not an option due to her poor lung function. She was dependent of others for washing, eating and toilet visits. She endured constant tiredness. She has not developed diabetes mellitus, but her HbA1c value was mildly elevated. This patient was emotionally labile. She decided to withdraw from periodic MRI scans of the breasts and lung function tests because these were considered too invasive, exhausting and painful. After several years, botulinum toxin injections partly relieved the pain in her neck. When she was 32 years old, she was bedridden because of a pressure sore.

Patient 7

This male patient had ataxia since he started to walk and was diagnosed with A-T at the age of 14 years. He became wheelchair bound at the age of 18 years. He did not have recurrent infections. ATM protein studies showed he had classic A-T with absence of ATM kinase activity. He lived with his mother and had to move to a nursing home when he was 46 years old, since his mother could not take care of him anymore. He developed urine incontinence and diabetes mellitus type 2. His lower extremities were paralysed. He could only lift his hands but was unable to lift his arms or move his fingers. He had contractures, antecollis of his head, and was poorly intelligible due to severe dysarthria (See *Online Resource 3*). Tube feeding was considered but not started because of co morbidity. He had pressure sores on his heels and buttock. Dental caries could not be treated due to a severe dysphagia and fear for complications after general anaesthesia. At the age of 50 years he was treated for haemorrhoids. He developed a persistent anaemia despite iron supplementation and erythrocyte transfusions. Gastroscopy showed a gastic antral vascular ectasia (‘watermelon stomach’) as a potential cause (see *Online Resource 2*). This was treated with argon-plasma coagulation. Furthermore, he was diagnosed with a prostate carcinoma that grew into his rectum. This was treated with hormonal therapy. At this point he did not want to undergo any further gastro-or colonoscopy. He died at his nursing home at the age of 54 years.
