# Supplementary material for: Classic ataxia-telangiectasia: the phenotype of long-term survivors
Source: J Neurol. 2019 Nov 27;267(3):830–7. doi: 10.1007/s00415-019-09641-1 (PMC7035236; doi:10.1007/s00415-019-09641-1)

Online Resource 2: Photos of patients 1 and 7.

Photo 1: Anteflexion of the head (patient 1).

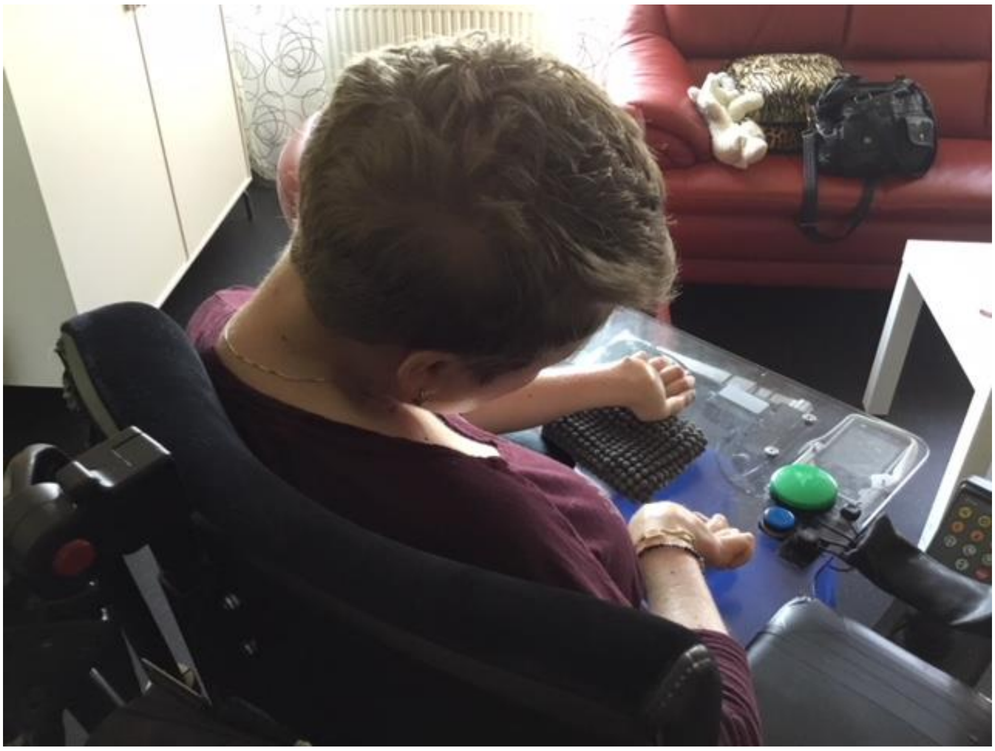

Photo 2: Contractures in the hands (patient 1).

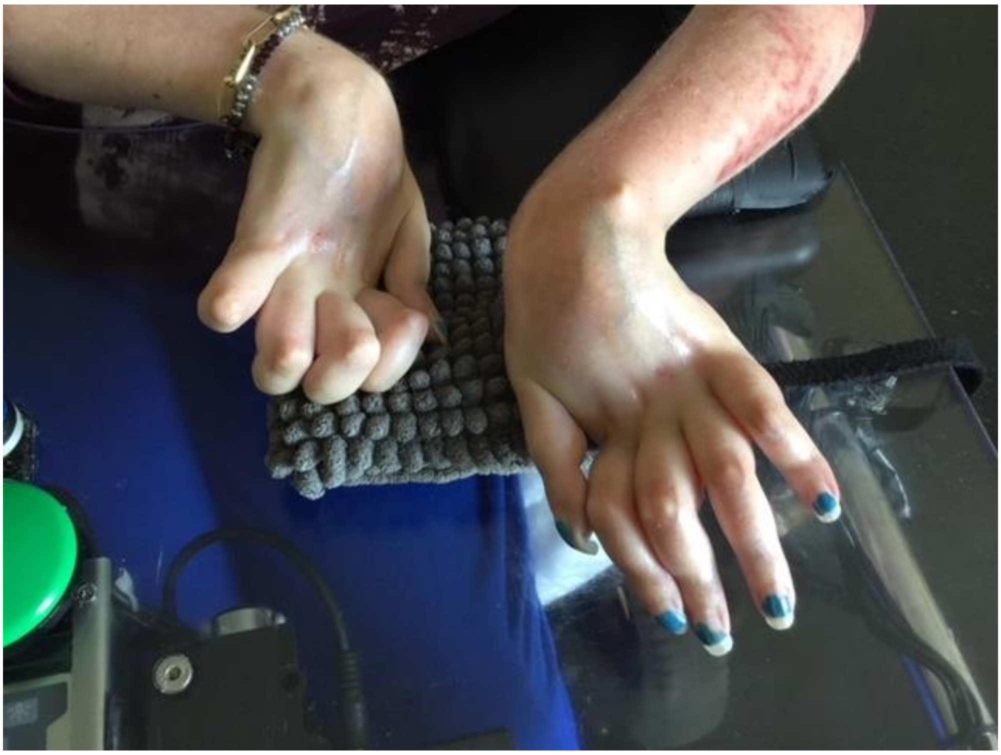

Photo 3: Foot drop with contractures (patient 1).

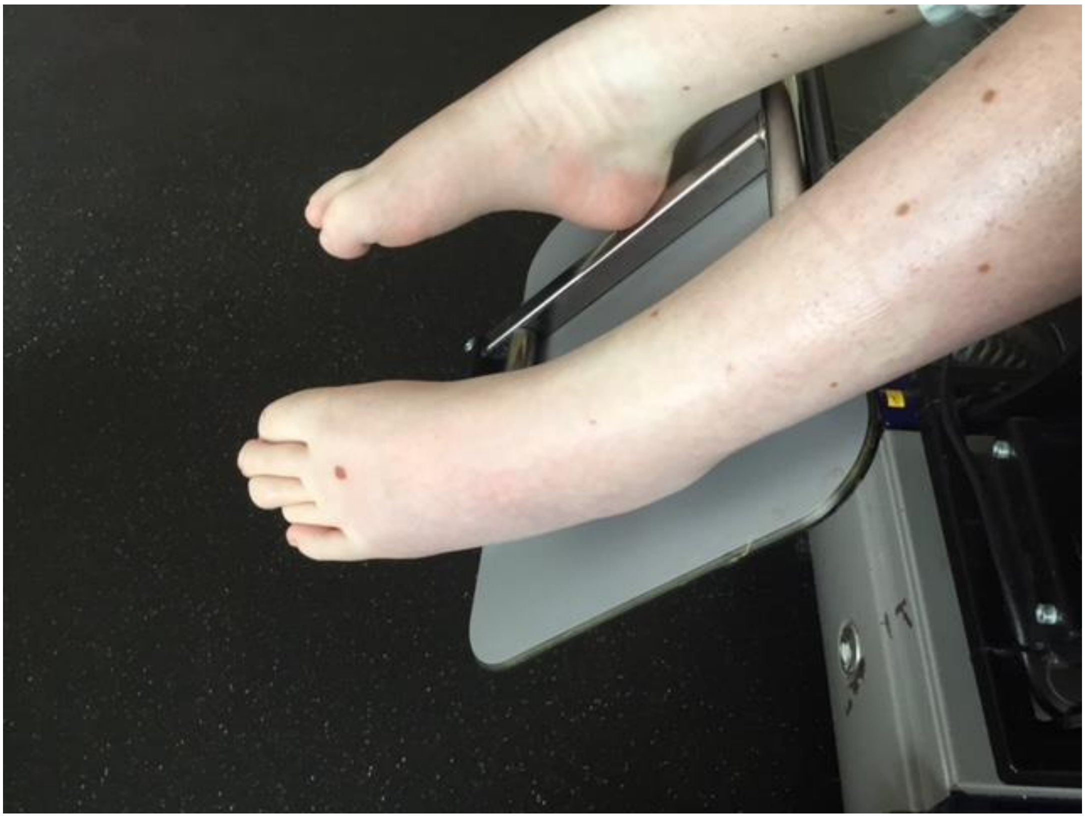

Photo 4: Gastric antral vascular ectasia (patient 7).

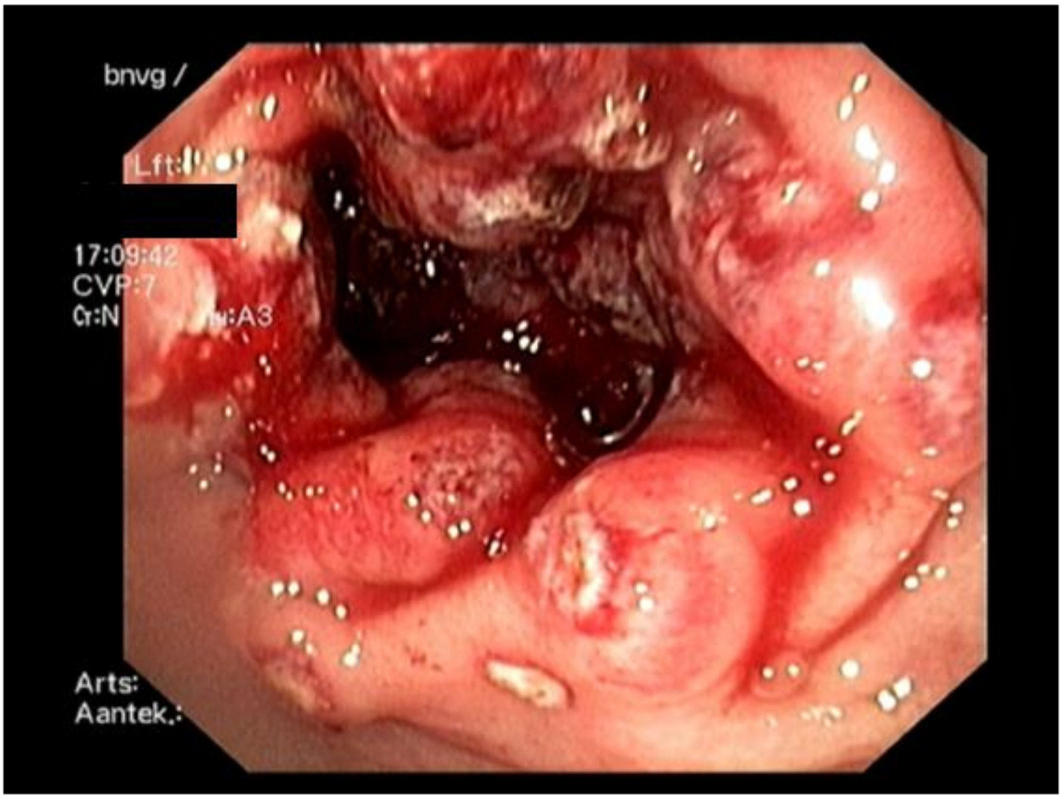

Supplement: Supplementary file 2 — Supplementary file2 (PDF 147 kb) [file 415_2019_9641_MOESM2_ESM.pdf]
