# Supplementary material for: Classic ataxia-telangiectasia: the phenotype of long-term survivors
Source: J Neurol. 2019 Nov 27;267(3):830–7. doi: 10.1007/s00415-019-09641-1 (PMC7035236; doi:10.1007/s00415-019-09641-1)
Supplement: Supplementary file 4 — Supplementary file4 (PDF 26 kb) [file 415_2019_9641_MOESM4_ESM.pdf]

Online Resource 4: Characteristics of 14 classic A-T patients from the literature.

	**Patient**	**Ataxia (years)**	**Wheelchair (years)**	**Immunodeficiency**	**Recurrent infections**	**Malignancy**	**Telangiectasias**	**AFP (ng/ml)**	**ATM mutations**	**Chromosomal rearrangements / radio sensitivity**
**Goodman 1969**	1	<1	10	Yes, IgA	Yes	x	Yes	x	x	x
	2	1	10	Yes, IgA	Yes	x	Yes	x	x	Yes
**Amromin 1977**		<1	9	Yes, IgA	Yes	Yes	Yes	1200	x	Yes
**Agamanolis 1979**		5	<20	No	Yes	No	Yes	x	x	Yes
**Cabot 1987**	Case 2	2	10	x	No	Yes	Yes	296	x	x
**Mock 1988**		Infan-cy	Child-hood	Yes, IgA	No	Yes	Yes	x	x	Yes
**Kovacs 1997**	Case 2	3	x	x	x	Yes	Yes	x	x	Yes
	Case 3	2	10	Yes	No	No	Yes	x	x	x
**Opeskin 1998**		x	x	x	Yes	Yes	Yes	x		Yes
**Degan 2007**	LA	x	x	Yes	Yes	x	Yes	High	Yes	x
**Habek 2008**		1	<10	Yes, IgG	Yes	Yes	Yes	518	x	Yes
**Lockman 2012**	E	x	Yes	x	x	Yes	Yes	x	Yes	x
	G	x	Yes	No	No	No	x	x	Yes	x
**Lin 2014**	9	x	x	x	x	x	x	484	x	Yes

x = not noted.
